# Towards a Quantitative Theory of Epidermal Calcium Profile Formation in Unwounded Skin

**DOI:** 10.1371/journal.pone.0116751

**Published:** 2015-01-27

**Authors:** Matthew P. Adams, Daniel G. Mallet, Graeme J. Pettet

**Affiliations:** 1 Mathematical Sciences School and Institute of Health and Biomedical Innovation, Queensland University of Technology, Brisbane, Queensland, Australia, and School of Chemical Engineering, The University of Queensland, Brisbane, Queensland, Australia; 2 Mathematical Sciences School and Institute of Health and Biomedical Innovation, Queensland University of Technology, Brisbane, Queensland, Australia; University Hospital Hamburg-Eppendorf, GERMANY

## Abstract

We propose and mathematically examine a theory of calcium profile formation in unwounded mammalian epidermis based on: changes in keratinocyte proliferation, fluid and calcium exchange with the extracellular fluid during these cells’ passage through the epidermal sublayers, and the barrier functions of both the stratum corneum and tight junctions localised in the stratum granulosum. Using this theory, we develop a mathematical model that predicts epidermal sublayer transit times, partitioning of the epidermal calcium gradient between intracellular and extracellular domains, and the permeability of the tight junction barrier to calcium ions. Comparison of our model’s predictions of epidermal transit times with experimental data indicates that keratinocytes lose at least 87% of their volume during their disintegration to become corneocytes. Intracellular calcium is suggested as the main contributor to the epidermal calcium gradient, with its distribution actively regulated by a phenotypic switch in calcium exchange between keratinocytes and extracellular fluid present at the boundary between the stratum spinosum and the stratum granulosum. Formation of the extracellular calcium distribution, which rises in concentration through the stratum granulosum towards the skin surface, is attributed to a tight junction barrier in this sublayer possessing permeability to calcium ions that is less than 15 nm s^−1^ in human epidermis and less than 37 nm s^−1^ in murine epidermis. Future experimental work may refine the presented theory and reduce the mathematical uncertainty present in the model predictions.

## Introduction

The calcium distribution within the mammalian epidermis is both an indicator of the skin barrier function [1] and a regulator of epidermal structure [2]. Here, using a mathematical model, we propose and examine a theory of the key mechanisms that control the calcium profile in unwounded epidermis.

### The epidermis and its calcium profile

The epidermis consists predominantly of keratinocytes [3]. These cells are continuously being produced at the bottom of the epidermis, driven to passively migrate towards the skin surface, and are sloughed away during everyday activity [4]. During this life cycle, keratinocytes express distinct phenotypic changes which characterise the boundaries of four sublayers of the epidermis:

1. The *stratum basale* (SB): Keratinocytes proliferate. The exact pattern of proliferation is still a matter of debate [5], and is suggested to involve either one [6, 7] or two cell types [8]. The single progenitor theory posits that a single population of slowly-cycling cells maintains epidermal homeostasis, whilst the more traditional two progenitor theory proposes that the SB consists of two keratinocyte subpopulations: (1) stem cells, which proliferate slowly and indefinitely, each time producing one stem cell and one transit amplifying (TA) cell, and (2) TA cells, which divide symmetrically 3–5 times before leaving the SB [9, 10].
2. The *stratum spinosum* (SS): Keratinocytes increase in volume [11] and passively migrate towards the skin surface, displaced from the SB by proliferation there.
3. The *stratum granulosum* (SG): Keratinocytes become flattened and disintegrate, reducing their volume [12] and expelling lamellar bodies [13].
4. The *stratum corneum* (SC): Denucleated and highly flattened keratinocytes, called corneocytes, combine with lipids from the lamellar bodies exocytosed in the SG, in a “bricks and mortar” architecture [14] that forms the primary skin barrier [4]. Transepidermal water loss (TEWL) experiments, which involve progressive tape-stripping of the SC to identify the thickness that must be removed to cause fluid flow to significantly increase across this sublayer, suggest that this barrier is only strongly impermeable in the top 4–8 *μ*m of the SC [15–17]. Hence we subdivide this epidermal sublayer into the *lower SC* (progressive barrier) and *upper SC* (impermeable barrier). At the top of the upper SC, intercorneocyte linking structures degrade and corneocytes are shed from the skin surface [18].

Epidermal calcium is present in three different localisations: the extracellular fluid (ECF), intracellular cytosol and intracellular organelles [19]. Calcium concentrations in the ECF and organelles are significantly higher than in the cytosol [20, 21]. These concentration differences are maintained by calcium pumps present on the membranes of keratinocytes and their intracellular structures, which actively remove calcium from the cytosol [22]. If we consider calcium in the ECF as “extracellular”, and calcium in cytosol and organelles together as “intracellular”, then it is the action of the calcium pumps on the keratinocyte membrane that is crucial for controlling intracellular and extracellular calcium levels [23].

**Figure 1:**
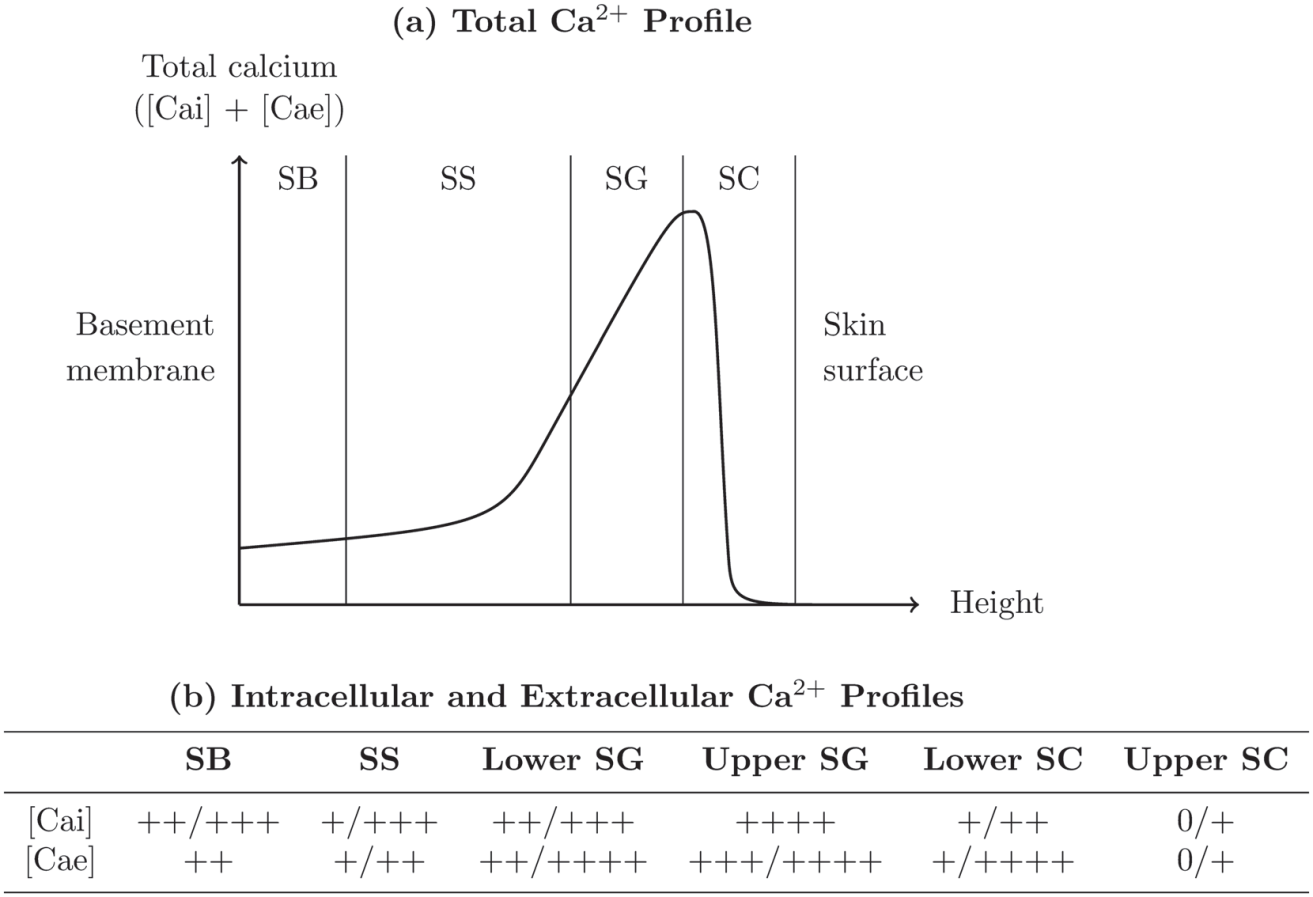
The epidermal calcium distribution. (a) Typical shape of the total profile found quantitatively using PIXE (for examples in the experimental literature, see [26, 28]). (b) Typical shape of the semi-quantitative intracellular ([Cai]) and extracellular ([Cae]) profiles measured using ion capture cytochemistry (for examples in the experimental literature, see [32–34]).

The total epidermal calcium profile, which is a summation of calcium from intracellular and extracellular localisations, has been quantitatively measured using proton-induced X-ray emission (PIXE) [24–28], and in unwounded skin these measurements typically adhere to the profile shown in Fig. 1a. The total calcium concentration is low in the SB, rises gradually to a peak in the SG (the so-called “epidermal calcium gradient”), and drops to near-negligible levels in the SC. Because the PIXE technique has a resolution of ∼ 10 *μ*m [29], it is unclear whether the calcium drop towards the skin surface occurs at the SG-SC interface or further into the SC: the latter interpretation is quite feasible since the skin’s primary barrier might only be fully formed in the upper SC, based on the previously discussed TEWL experiments. PIXE cannot distinguish between the intracellular and extracellular contributions to the epidermal calcium profile.

On the other hand, the intracellular and extracellular epidermal calcium profiles have been measured separately using ion capture cytochemistry [30, 31], but only semi-quantitatively [32–34]. As indicated in Fig. 1b, both intracellular and extracellular profiles qualitatively agree with the total profiles obtained from PIXE, but it is difficult to make additional interpretations from this semi-quantitative data.

For the past decade, the presence of the epidermal calcium profile has been attributed solely to the presence of the SC barrier [35], which is thought to act as a sieve, selectively allowing water but not calcium to leave the viable epidermis [36]. When the epidermis is wounded, its calcium profile disappears rapidly then reappears gradually with restoration of the skin’s barrier function [1, 37, 38]. This observation fits easily within the conventional sieve view of epidermal calcium profile formation, as the removal of the SC simply removes the impetus for the calcium gradient to form.

However, recent measurements of the epidermal calcium distribution using fluorescent lifetime imaging [36, 39] have brought this view into question. These measurements demonstrated that the bulk of free calcium is present in intracellular organelles [36], and that epidermal barrier disruption triggers a mobilization of high amounts of calcium from these stores [39]. This prompted the questioning of this conventional view that the epidermal calcium profile is regulated only passively by the SC. In previous work, using a mathematical model, we found that this profile is largely intracellular and regulated by sublayer-specific changes in the action of keratinocyte membrane pumps [23]. In the current paper, we extend this analysis further, to propose that there are three key mechanisms that control epidermal calcium profile formation in unwounded skin: the passive impermeable barrier of the SC, tight junction-limited calcium diffusion in the SG, and a phenotypic switch in calcium exchange between keratinocytes and extracellular fluid at the SS-SG boundary. We also investigate the contribution of the stem and TA cell subpopulations of the SB, volume changes of keratinocytes in the SS, and calcium located in the lower SC, to the formation of the calcium profile of unwounded epidermis.

### Proposed key mechanisms regulating the calcium profile

**Figure 2:**
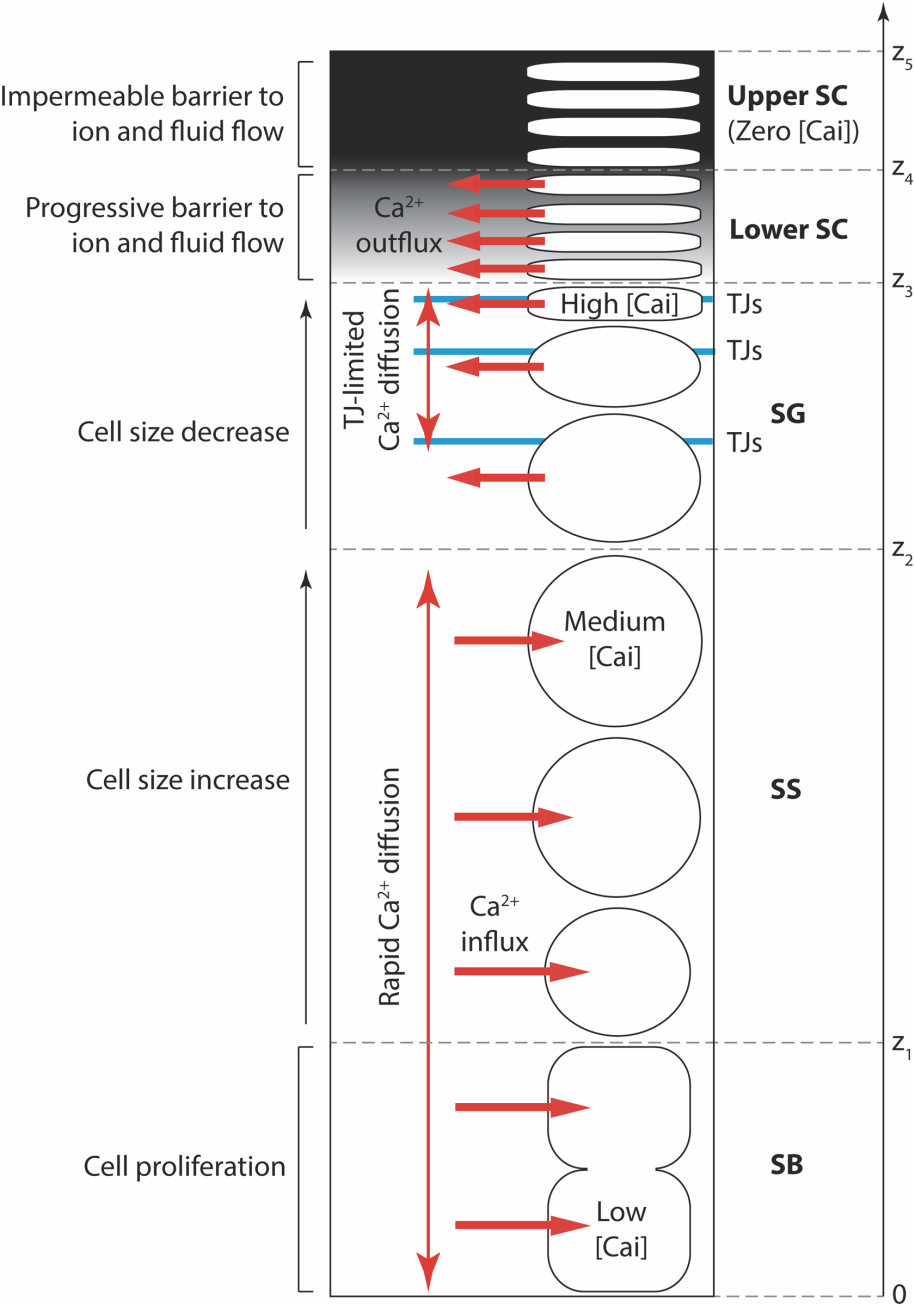
Proposed conceptual model of epidermal calcium profile formation in unwounded skin. The mathematical model presented in this paper simplifies the progressive barrier in the lower SC to a distinct barrier at the lower-upper SC boundary.

Our proposed theory is presented schematically in Fig. 2. We treat the calcium present in the cytosol and organelles within keratinocytes together as intracellular calcium, with the majority of this calcium likely to be confined to the keratinocyte organelles [21]. Most epidermal calcium is present in this intracellular calcium [36], which possesses a distinct spatial profile that forms as follows. Membrane pumps on keratinocytes act to accumulate calcium intracellularly from the ECF in the SB and SS, and in the SG this behaviour reverses to calcium expulsion into the ECF [23], emptying the intracellular stores [39] so that corneocytes in the upper SC contain negligible levels of intracellular calcium. These mechanisms yield an intracellular calcium profile that is low in the SB, rises gradually towards a peak in the SG, and drops rapidly in the SC, in agreement with the experimental observations for both the total and intracellular profiles (see Figs. 1a and 1b).

The extracellular calcium profile, which possesses far less calcium due to the small volume of the epidermis occupied by the ECF [36, 40], forms as follows. The ECF is essentially water [41], and hence extracellular calcium in the SB and SS diffuses rapidly to near-constant levels throughout these sublayers [23]. In the SG, cell-cell adhesions known as tight junctions (TJs) are located apically between the lateral membranes of neighbouring keratinocytes [42, 43], and form a permeability barrier to calcium ions [44, 45] that reduces the rate of extracellular calcium diffusion there. Because calcium is continuously being expelled by keratinocytes near the skin surface, this TJ-limited calcium diffusion in the SG causes the extracellular calcium concentration to be slightly elevated there, negligibly affecting the calcium levels in the underlying SB and SS [46]. Lipids cannot be responsible for this elevated extracellular calcium concentration in the SG because they are localised only at the SG-SC boundary prior to their contribution as the “mortar” of the SC barrier. Extracellular calcium cannot enter the upper SC due to its barrier function, in agreement with the TEWL experiments [15–17]. These mechanisms together yield an extracellular calcium profile which is nearly constant in the SB and SS, rises in the SG, and drops rapidly in the SC, in agreement with experimental observations of the extracellular profile (see Fig. 1b).

## Materials and Methods

### Main equations

We mathematically model the epidermis as a saturated porous medium [47]. This modelling strategy has been used previously to consider avascular tumour growth [48–50] and cell behaviour within an artificial scafffold [51], justified for the viable sublayers of the epidermis in our previous paper [23], and proposed for modelling the SC of the epidermis by Kitson and Thewalt [52].

As a porous medium, we assume that the keratinocytes behave uniformly and are analogous to soil particles, and the surrounding ECF is analogous to the water that saturates the soil system. We assume that keratinocytes and ECF are comprised of an identical, incompressible fluid. Calcium is always dissolved in the cells or ECF. Calcium contained in the cytosol and intracellular organelles of cells are considered together simply as intracellular calcium. This simplification means that we do not specifically consider the intracellular dynamics of calcium exchange between the cytosol and organelles. We cannot discount the possibility that the intracellular calcium dynamics may play an important role in the partitioning of calcium between intracellular and extracellular domains, although investigating this is beyond the scope of the present work. As we are only interested here in identifying the extracellular and intracellular contributions to the epidermal calcium profile, consideration of cytosolic and organelle calcium separately is not necessary to investigate our proposed theory. Experimentally, intracellular calcium waves are known to propagate between adjacent keratinocytes [53], but these waves negligibly affect the epidermal calcium profile. Hence, in our model calcium cannot travel directly between keratinocytes, but rather can only be exchanged between cells and the surrounding ECF.

We assume that both the structure and calcium profile of the epidermis have reached a distribution that is stable and unchanging with time. Because of this we consider only one spatial direction *z* perpendicular to the skin surface. For this simplification, we ensured that any model parameters recorded for the three-dimensional case are also appropriate for the one-dimensional case. The main equations of our model, derived from mass conservation equations for the fluid and calcium present both in cells and ECF, are identical to those from our previous paper [23], but with one important exception. We do not specify the ECF velocity, because it will be unpredictably modified by TJs [54] and aquaporins [55, 56], neither of which were considered in [23]. With all these considerations in mind, the main equations of our model are

$$\frac{\text{d}}{\text{d}z}\left(\phi u_{i}\right)=f,$$ (1a)

$$\frac{\text{d}}{\text{d}z}\left(\rho_{ci}u_{i}\right)=g,$$ (1b)

$$\frac{\text{d}}{\text{d}z}\left(\rho_{ce}u_{ce}\right)=-g,$$ (1c)

where *φ* is the cell volume fraction, *ρ*_*ci*_ and *ρ*_*ce*_ are the superficial intracellular and extracellular calcium concentrations respectively, *u*_*i*_ and *u*_*ce*_ are the physical velocities of the cells and extracellular calcium respectively, *f* is the rate of change of cell volume fraction due to fluid exchange between ECF and cells, and *g* is the rate of change of superficial intracellular calcium concentration due to calcium exchange between ECF and cells. Functions *f* and *g* are positive when fluid and calcium respectively are being transferred from ECF to cells, and negative when fluid and calcium respectively are being transferred from cells to ECF. We next use equations (1a)–(1c), together with defined boundary conditions, to derive equations for calculating: keratinocyte velocity profiles *u*_*i*_(*z*) and transit times through the epidermis, the intracellular calcium profile *ρ*_*ci*_(*z*) and pattern of calcium exchange between keratinocytes and the ECF *g*(*z*), and the dependence of the extracellular calcium profile *ρ*_*ce*_(*z*) on the permeability of the TJ barrier to calcium ions.

### Model domain and boundary conditions

In this section, we define the model domain and provide two boundary conditions each for *u*_*i*_(*z*), *ρ*_*ci*_(*z*) and *ρ*_*ce*_(*z*) as part of our proposed theory, although not all of these conditions will be necessary for our subsequent analysis. The epidermal sublayers shown in Fig. 2 are defined as follows: the SB in 0 ≤ *z* ≤ *z*_1_, the SS in *z*_1_ < *z* ≤ *z*_2_, the SG in *z*_2_ < *z* ≤ *z*_3_, the lower SC in *z*_3_ < *z* ≤ *z*_4_ and the upper SC in *z*_4_ < *z* ≤ *z*_5_. We assume that the two progenitor theory holds for human and murine epidermis [8]. In the two progenitor theory, the SB consists of stem cell and TA cell subpopulations which are suggested to form two spatially separate compartments [57, 58]. Hence we subdivide the SB into compartments consisting of stem cells, 0 ≤ *z* ≤ *θz*_1_, and TA cells, *θz*_1_ < *z* ≤ *z*_1_, where *θ* is the volume fraction of the SB occupied by stem cells.

In our model, equation (1a) defines the dynamics of epidermal cells, whilst equations (1b) and (1c) define the dynamics of epidermal calcium. Because keratinocytes occupy all sublayers of the epidermis, the model domain for equation (1a) is 0 ≤ *z* ≤ *z*_5_. Keratinocytes cannot pass through the BM (*z* = 0) but are continuously expelled at the skin surface (*z* = *z*_5_), sloughed away during everyday activity [4]. Hence the boundary conditions for equation (1a) are

$$u_{i}(0)=0,$$ (2a)

$$u_{i}(z_{5})>0.$$ (2b)

Our description of epidermal calcium profile formation treats the lower SC as a progressive barrier and the upper SC as an impermeable barrier to fluid and ion flow, based on TEWL experiments [15–17] and the observation of non-negligible calcium levels in the lower SC [33]. In our model we simplify this to treat the boundary between the lower and upper SC, denoted *z*_4_, as the impermeable barrier to transport of fluid and ions. Hence the model domain for equations (1b) and (1c) is 0 ≤ *z* ≤ *z*_4_.

Intracellular calcium cannot travel across the BM because it is contained within keratinocytes, and is completely absent in the corneocytes of the upper SC [34, 37]. Hence the boundary conditions for equation (1b) are

$$\rho_{ci}(0)u_{i}(0)=0,$$ (2c)

$$\rho_{ci}(z_{4})=0.$$ (2d)

The calcium present in the epidermis originates from movement of fluids and calcium across the BM [59], which at steady state must therefore act as a source of extracellular calcium with constant and positive concentration. Extracellular calcium is prevented from entering the upper SC by the impermeable barrier acting at *z*_4_. Hence the boundary conditions for equation (1c) are

$$\rho_{ce}(0)=0,$$ (2e)

$$\rho_{ce}(z_{4})u_{ce}(z_{4})=0.$$ (2f)

For the analysis performed in this paper, we will only explicitly require two of the six boundary conditions listed here, equations (2a) and (2f).

### Calculating keratinocyte velocity profiles and transit times

Using equation (1a), the keratinocyte velocity profile *u*_*i*_(*z*) is estimated from profiles that we now define for the cell volume fraction, *φ*(*z*), and volume exchange between cells and ECF, *f*(*z*). We specify *f*(*z*) as

$$f(z)=\{\begin{array}{ll} s_{0}\phi , & 0\leq z\leq \theta z_{1},\\ s_{1}\phi , & \theta z_{1}<z\leq z_{1},\\ s_{2}\phi , & z_{1}<z\leq z_{2},\\ -s_{3}\phi , & z_{2}<z\leq z_{3},\\ 0, & z_{3}<z\leq z_{5}. \end{array}$$ (3)

This form expresses the different proliferation rates *s*_0_ and *s*_1_ of stem and TA cells in the SB [60], the rate of volume increase *s*_2_ for keratinocytes migrating through the SS [11], the rate of volume decrease *s*_3_ for keratinocytes migrating through the SG [12], and the relative structural inertness of corneocytes in the SC [61].

The cell volume fraction *φ* is assumed to be constant and equal to *φ*_*v*_ throughout both the viable sublayers (SB, SS and SG) and the lower SC [62]. The “bricks and mortar” architecture of the upper SC [14] constitutes a slow-moving relatively impenetrable barrier to fluid transport [63], equivalent to a sublayer consisting solely of keratinocyte-derived contents (*φ* = 1). Hence the cell volume fraction profile *φ*(*z*) is specified as

$$\phi (z)=\{\begin{array}{ll} \phi_{v}, & 0\leq z\leq z_{4},\\ 1, & z_{4}<z\leq z_{5}. \end{array}$$ (4)

The superficial keratinocyte velocity *φu*_*i*_ is assumed to be continuous at each of the sublayer boundaries, to ensure that cell mass flow is continuous throughout the epidermis. This consideration, together with equations (1a), (2a), (3) and (4), yield the keratinocyte velocity profile *u*_*i*_(*z*) as

$$u_{i}(z)=\{\begin{array}{ll} s_{0}z, & 0\leq z\leq \theta z_{1},\\ u_{i}(\theta z_{1})+s_{1}(z-\theta z_{1}), & \theta z_{1}\leq z\leq z_{1},\\ u_{i}(z_{1})+s_{2}(z-z_{1}), & z_{1}\leq z\leq z_{2},\\ u_{i}(z_{2})-s_{3}(z-z_{2}), & z_{2}\leq z\leq z_{3},\\ u_{i}(z_{3}), & z_{3}\leq z\leq z_{4},\\ \phi_{v}u_{i}(z_{4}), & z_{4}<z\leq z_{5}. \end{array}$$ (5)

Rates *s*_2_ and *s*_3_ are obtained from empirical observations of the ratio of keratinocyte volumes between the upper and lower boundaries of the SS, *V*_1_ > 1 (net volume increase from lower to upper boundary), and the SG, *V*_2_ < 1 (net volume decrease from lower to upper boundary), respectively, by use of the equations

$$s_{2}=\frac{u_{i}(z_{1})}{z_{2}-z_{1}}(V_{1}-1),$$ (6a)

$$s_{3}=\frac{u_{i}(z_{2})}{z_{3}-z_{2}}(1-V_{2}).$$ (6b)

Equations (6a) and (6b) can be obtained using mathematical procedures similar to the derivation of *s*_2_(*R*) provided in Appendix B of [23].

Using the cell velocity profiles *u*_*i*_(*z*) defined by equations (5), (6a) and (6b), transit times through the various epidermal sublayers are calculated via

$$\tau (z_{a},z_{b})=\int_{z_{a}}^{z_{b}}\frac{\text{d}z}{u_{i}(z)},$$ (7)

where *τ*(*z*_*a*_,*z*_*b*_) is the average time taken for a keratinocyte to move from height above the BM *z*_*a*_ to height *z*_*b*_. We assume that the transit through the SB can be approximated by the transit through the TA cell compartment, because the volume of SB occupied by stem cells is negligible compared to TA cells [64], and stem cells possess theoretically infinite transit time because they may never leave the SB. Hence, from equations (5) and (7) the epidermal transit times are given by

$$\tau_{\text{SB}}\approx \tau (z_{0},z_{1})=\frac{1}{s_{1}}\ln\left(\frac{u_{i}(z_{1})}{u_{i}(z_{0})}\right),$$ (8a)

$$\tau_{\text{SS}}=\tau (z_{1},z_{2})=\frac{1}{s_{2}}\ln(V_{1}),$$ (8b)

$$\tau_{\text{SG}}=\tau (z_{2},z_{3})=-\frac{1}{s_{3}}\ln(V_{2}),$$ (8c)

$$\tau_{\text{SC}}=\tau (z_{3},z_{5})=\frac{1}{u_{i}(z_{3})}\left(z_{4}-z_{3}+\frac{z_{5}-z_{4}}{\phi_{v}}\right).$$ (8d)

### Calculating profiles of intracellular calcium and calcium exchange

In this section we show how the intracellular calcium profile *ρ*_*ci*_(*z*) and calcium exchange between keratinocytes and ECF *g*(*z*), can be estimated from the total epidermal calcium profile *ρ*(*z*).

The total calcium profile is a summation of intracellular and extracellular calcium profiles,

$$\rho (z)=\rho_{ci}(z)+\rho_{ce}(z),$$ (9)

but extracellular calcium provides only a small contribution (2–10 mg/kg) to the total calcium profile in the epidermis (100–1100 mg/kg) [23, 36]. Hence, to estimate the intracellular calcium profile *ρ*_*ci*_(*z*) from the total calcium profile *ρ*(*z*) using equation (9), at the scale of *ρ*(*z*) we approximate the extracellular calcium distribution by a constant equal to its mean value throughout the epidermis,

$$\rho_{ce}(z)\approx r\rho_{ce}(0).$$ (10)

Here, *r* is a nondimensional factor equal to the ratio of the mean extracellular calcium concentration of all sublayers enclosed by [0, *z*_4_] to its concentration at the BM, and whose uncertainty bounds express the variation of the extracellular calcium concentration throughout these sublayers. The BM levels of total and extracellular calcium are related by

$$\rho (0)=\frac{\rho_{ce}(0)}{1-\phi_{v}},$$ (11)

an equation that was derived in Appendix C of [23] under two assumptions: (1) the motion of calcium across the BM only involves transfer between the free dermal and extracellular epidermal calcium, and (2) the BM provides no barrier for this transfer.

Combining equations (9)–(11), the intracellular calcium profile can be estimated from the total calcium profile via

$$\rho_{ci}(z)\approx \rho (z)-r(1-\phi_{v})\rho (0).$$ (12)

Equations (5) and (12) can be used to estimate the keratinocyte velocity profile *u*_*i*_(*z*) and intracellular calcium profile *ρ*_*ci*_(*z*). The pattern of calcium exchange *g*(*z*) between cells and ECF can then be calculated from these two profiles using equation (1b) [23],

$$g(z)=\frac{\text{d}}{\text{d}z}(\rho_{ci}(z)u_{i}(z)).$$

In the following, we derive equations that link the extracellular calcium distribution to the permeability of the TJ barrier.

### The effect of tight junctions on extracellular calcium diffusion

TJs regulate the extracellular flow of calcium ions in the SG [44, 45], and we model this as a reduction in the rate of extracellular calcium diffusion there. This effect is introduced through the term representing extracellular calcium flux, *ρ*_*ce*_ *u*_*ce*_, that appears in equation (1c). The extracellular calcium flux *ρ*_*ce*_ *u*_*ce*_ may consist of contributions from both diffusion and advection, the latter of which we expect to be negligible in epidermal sublayers where TJs are not present [23]. However, in epidermal sublayers where TJs are present, for advection to be negligible compared to diffusion we must ensure explicitly that the Péclet number, Pe, satisfies

$$\text{Pe}=\frac{\hat{z}|u_{e}|}{D}\ll 1,$$ (13)

where $\hat{z}\leq z_{4}$ is the characteristic length scale over which the effects of diffusion and advection are being compared, ∣*u*_*e*_∣ is the ECF velocity that characterises the advective contribution, and *D* is the Fickian diffusion coefficient that characterises the diffusive contribution. In this paper we limit our analysis to cases for which inequality (13) is satisfied. We specify the extracellular calcium diffusion coefficient as

$$D(z)=\{\begin{array}{ll} D_{\text{Ca}}, & 0\leq z\leq z_{2},\\ \varepsilon_{\text{Ca}}D_{\text{Ca}}, & z_{2}<z\leq z_{3},\\ D_{\text{Ca}}, & z_{3}<z\leq z_{4}, \end{array}$$ (14)

where *D*_Ca_ is the physical diffusion coefficient of calcium in the ECF in the absence of TJs, and *ε*_Ca_ represents the factor reduction in diffusion coefficient *D*_Ca_ induced by the presence of TJs.

In equation (14) we have assumed that TJs are evenly spread throughout the SG, which represents a simplification to the dynamic model we proposed for skin equivalent construct growth [46, 65], and that they are mostly absent in other sublayers. Whilst structures similar to the disassembly of TJs have been observed at the SG-SC interface [66] and TJ-like structures have been observed in the SC [67], for simplicity we assume that these structures provide no restriction on extracellular calcium ion flow there.

The permeability of a barrier can be written as a ratio of the diffusion coefficient of the substance within the barrier to the barrier’s width [68]. Hence the permeability of the TJ barrier to calcium, *P*_Ca_, which spans the SG *z*_2_ to *z*_3_, and has local diffusion coefficient there of *ε*_Ca_ *D*_Ca_ according to equation (14), is

$$P_{\text{Ca}}=\frac{\varepsilon_{\text{Ca}}D_{\text{Ca}}}{z_{3}-z_{2}}.$$ (15)

Combining equations (13)–(15), we find that the inequality

$$P_{\text{Ca}}\gg |u_{e}|,$$ (16)

is identical to the requirement given by inequality (13). Inequality (16) demonstrates that the permeability of the TJ barrier must be significantly larger than the local ECF velocity in order to disregard the contribution of advection to extracellular calcium dynamics. From [23] we expect that max{∣*u*_*e*_∣} is $O(1\;\mathrm{nm}s^{-1})$ in the absence of TJs and aquaporins and hence we require

$$P_{\text{Ca}}\gg O(1\;\text{nm}s^{-1}),$$ (17)

which effectively places a lower limit on the possible values of *P*_Ca_ that we investigate here. In summary, we include the effect of tight junctions on extracellular calcium dynamics in our model by assuming that the extracellular calcium flux *ρ*_*ce*_ *u*_*ce*_ in equation (1c) is dominated by Fickian diffusion with coefficient *D* defined by equation (14), and this approach is valid if the permeability of the TJ barrier in the SG satisfies inequality (17).

### Calculating the extracellular calcium profile

To derive an expression for the extracellular calcium profile *ρ*_*ce*_(*z*), we first equate (1b) and (1c) through the common term *g*, and assume that Fickian diffusion is the dominant contribution to the extracellular calcium flux, *ρ*_*ce*_ *u*_*ce*_ = −*D* d*ρ*_*ce*_/d*z*, to obtain

$$\frac{\text{d}}{\text{d}z}(\rho_{ci}u_{i})=\frac{\text{d}}{\text{d}z}\left(D\frac{\text{d}\rho_{ce}}{\text{d}z}\right).$$ (18)

Both sides of equation (18) are then integrated with limits *z* and *z*_4_. We thereafter substitute boundary condition (2f), which yields

$$\frac{\text{d}\rho_{ce}}{\text{d}z}(z)=\frac{1}{D(z)}\left(\rho_{ci}(z)u_{i}(z)-\rho_{ci}(z_{4})u_{i}(z_{4})\right).$$ (19)

In epidermal sublayers where TJs are not present (i.e. everywhere except the SG), extracellular calcium kinetics are sufficiently dominated by diffusion that *ρ*_*ce*_ is constant [23]. Hence, replacing *z* by *z* ^′^ in equation (19), integrating this equation with limits 0 and *z*, and substituting equations (14) and (15), yields

$$\rho_{ce}(z)=\{\begin{array}{ll} \rho_{ce}(0), & 0\leq z\leq z_{2},\\ \rho_{ce}(0)+\int_{z_{2}}^{z}\frac{\rho_{ci}(z^{\prime })u_{i}(z^{\prime })-\rho_{ci}(z_{4})u_{i}(z_{4})}{(z_{3}-z_{2})P_{\text{Ca}}}\text{d}z^{\prime }, & z_{2}\leq z\leq z_{3},\\ \rho_{ce}(z_{3}), & z_{3}<z\leq z_{4}. \end{array}$$ (20)

In this equation, *ρ*_*ci*_(*z*) can be calculated from *ρ*(*z*) using equation (12). Hence, equation (20) expresses the extracellular calcium profile *ρ*_*ce*_(*z*) in terms of *ρ*(*z*), *u*_*i*_(*z*) and *P*_Ca_, if inequality (17) is satisfied.

### Relationship between tight junctions and the extracellular calcium profile

Finally, to clearly demonstrate the effect of the TJ barrier on the extracellular calcium profile, we define *R*_*ce*_ as the rise in extracellular calcium through the SG,

$$R_{ce}=\frac{\rho_{ce}(z_{3})}{\rho_{ce}(z_{2})}.$$ (21)

From equations (20) and (21), the relationship between the rise in extracellular calcium concentration through the TJ barrier in the SG, *R*_*ce*_, and the permeability of this barrier, *P*_Ca_, can be written in the elegant form

$$R_{ce}=1+\frac{P_{0}}{P_{\text{Ca}}},$$ (22)

where *P*_0_ is a constant that depends on the epidermal keratinocyte velocity and calcium profiles,

$$P_{0}=\frac{1}{\left(z_{3}-z_{2})\rho_{ce}(0\right)}\int_{z_{2}}^{z_{3}}\left(\rho_{ci}(z)u_{i}(z)-\rho_{ci}(z_{4})u_{i}(z_{4})\right)\text{d}z.$$ (23)

Using equations (22) and (23), the effects of a range of values for the permeability of the TJ barrier to calcium *P*_Ca_ on the defining feature of the extracellular calcium profile (its rise through the SG, *R*_*ce*_) can be easily investigated, once the value of *P*_0_ is known.

## Results

The key predictions of our model are presented here. All mathematical equations were stated and derived in Materials and Methods. All parameters were obtained from experimental literature (see S1 Text) and are stated in Table 1. In our calculations we also used the total calcium profiles *ρ*(*z*) for human and murine epidermis reported in [28] and [26] respectively. All uncertainty bounds were calculated using error propagation formulae from [69, 70] under the assumption that the error distributions of all parameters were independent (i.e. zero covariance).

**Table 1:** Model Parameters.

**Parameter**	**Value and Reference**	
	**Human**	**Murine**
Stem cell volume fraction of the SB, *θ*	0.055±0.045 [64]	0.055±0.045 [64]
Height of the SB-SS boundary above the BM, *z*_1_	45 *μ*m [95]	20 *μ*m [26]
Height of the SS-SG boundary above the BM, *z*_2_	75 *μ*m [95]	60 *μ*m [26]
Height of the SG-SC boundary above the BM, *z*_3_	105 *μ*m [95]	90 *μ*m [26]
Height of the inner SC-outer SC boundary above the BM, *z*_4_	118.5±1.5 *μ*m [15–16, 28]	94±2 *μ*m [17, 26]
Thickness of the epidermis, *z*_5_	125 *μ*m [28]	100 *μ*m [26]
Ratio of keratinocyte volumes SG:SB, *V*_1_	1.9±0.5 [96]	2.8±1.4 [97, 98]
Ratio of keratinocyte volumes SC:SG, *V*_2_	0.54±0.10 (original) [12] 0.100±0.026 (modified) [96, 99]	0.068±0.03 [97–99]
Proliferation rate of stem cells in the SB, *s*_0_	5.6×10^−7^ s^−1^ [60]	1.4×10^−6^ s^−1^ [60]
Proliferation rate of TA cells in the SB, *s*_1_	(1.7±1.1)×10^−6^ s^−1^ [88, 100]	(2.8±1.3)×10^−6^ s^−1^ [101]
Physical diffusion coefficient of calcium in the ECF, *D*_Ca_	1×10^−9^ m^2^ s^−1^ [102–104]	1×10^−9^ m^2^ s^−1^ [102–104]
Cell volume fraction in viable epidermis and lower SC, *φ*_*v*_	0.955±0.025 [36]	0.9925±0.0025 [40]
Ratio of the extracellular calcium distribution to its BM value, *r*	1.1±0.6 [33, 34]	1.25±0.75 [32]

Parameters used for the numerical solutions in this paper. Justification is provided in S1 Text.

### Epidermal transit times and keratinocyte velocities

**Figure 3:**
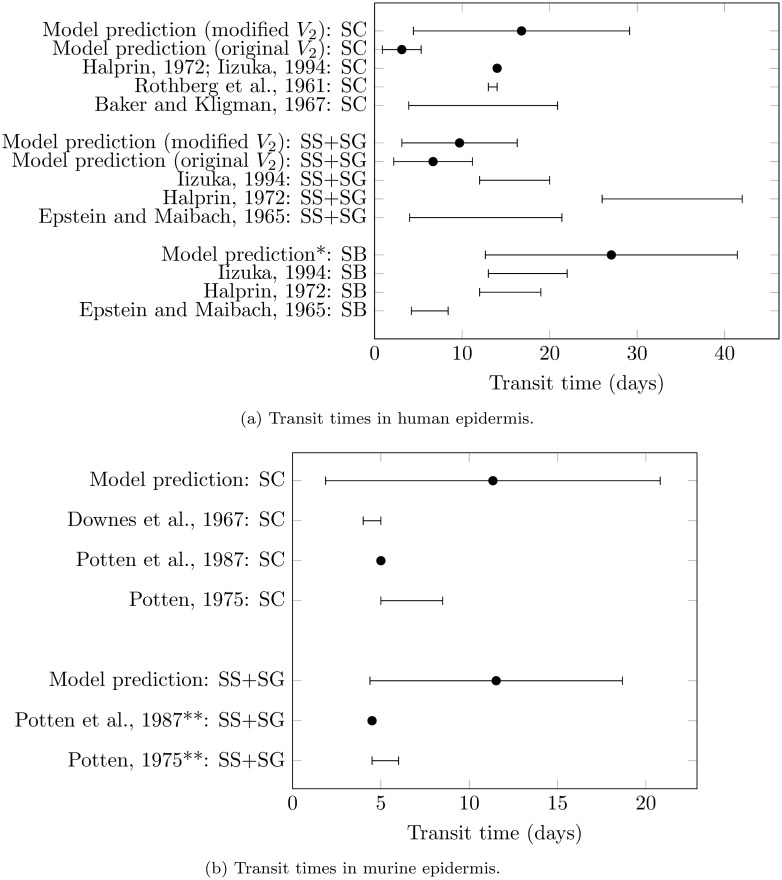
Comparison of epidermal sublayer transit times predicted by our model with experimental literature values. (a) Human literature values from [87–91]. (b) Murine literature values from [92–94]. *Model prediction in the SB was independent of the value of *V*_2_. **Value may include some residence time in the SB.

Using equations (5)–(8) of our model, transit times through individual sublayers of human and murine epidermis were calculated. Our model’s predictions of transit times mostly compared favourably with the literature values, as shown in Fig. 3, although it is difficult to quantitatively compare these values due to the large uncertainty present in the transit times both from the literature and predicted by our model. The uncertainty in our model predictions of transit time is due to the uncertainty present in model parameters (Table 1), all of which were obtained from the experimental literature. Hence, a better quantitative comparison of transit times from the literature and model requires experimental data possessing reduced uncertainty. We could not find literature values of transit time through murine SB so did not include comparisons for these.

The model prediction of transit time through human SC was much smaller than two of the three corresponding literature estimates. We attributed this discrepancy to our parameter estimate for human *V*_2_ = 0.54±0.10, which was much larger than the estimate for murine *V*_2_ = 0.068±0.034, the latter of which led to reasonable predictions of murine transit times. Hence, we modified our estimate of human *V*_2_ to 0.100±0.026, a value which was calculated from division of literature values for murine *V*_1_×*V*_2_ by human *V*_1_ (see S1 Text). The resulting predicted transit time for human SC agreed far better with the literature values for this transit time (Fig. 3a). Because this modification of *V*_2_ created agreement between estimates of keratinocyte volume size changes and transit times through our model, our analysis suggests that keratinocytes lose at least 87% of their volume during their disintegration in the SG, in both human and murine epidermis.

**Figure 4:**
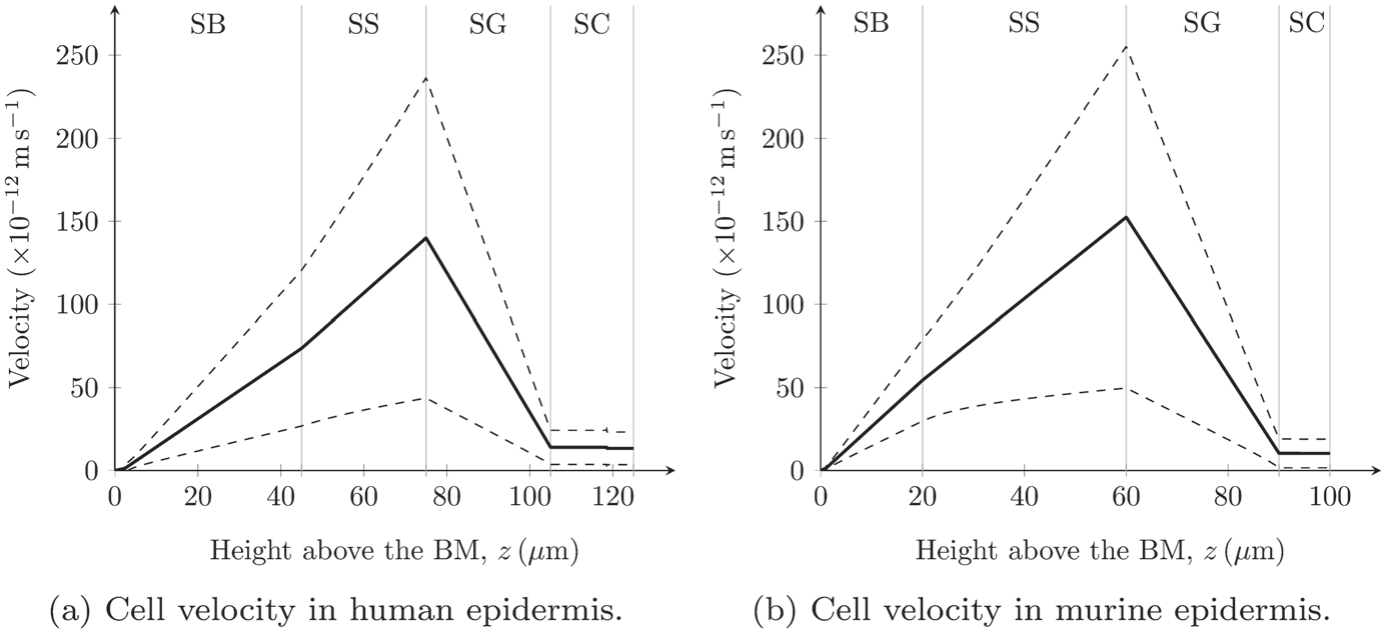
Keratinocyte velocity profiles predicted by our model. For (a) the human keratinocyte velocity profile, the modified *V*_2_ = 0.100±0.026 was used in its calculation. The solid and dashed lines represent the mean values and uncertainty bounds (± SD) respectively.

Keratinocyte velocity profiles *u*_*i*_(*z*) calculated using equations (5), (6a) and (6b) are shown in Fig. 4. For the calculation of the human *u*_*i*_(*z*) profile, the modified *V*_2_ was used. Regardless of the value of human *V*_2_, in our model results there was little difference between the keratinocyte velocity distributions in the lower sublayers of human and murine epidermis. This conclusion extends to the upper sublayers if the keratinocyte volume decrease through human SG agrees with our modified value for *V*_2_ (i.e. 90.0±2.6% volume reduction).

### The extracellular calcium rise mediated by tight junctions

**Figure 5:**
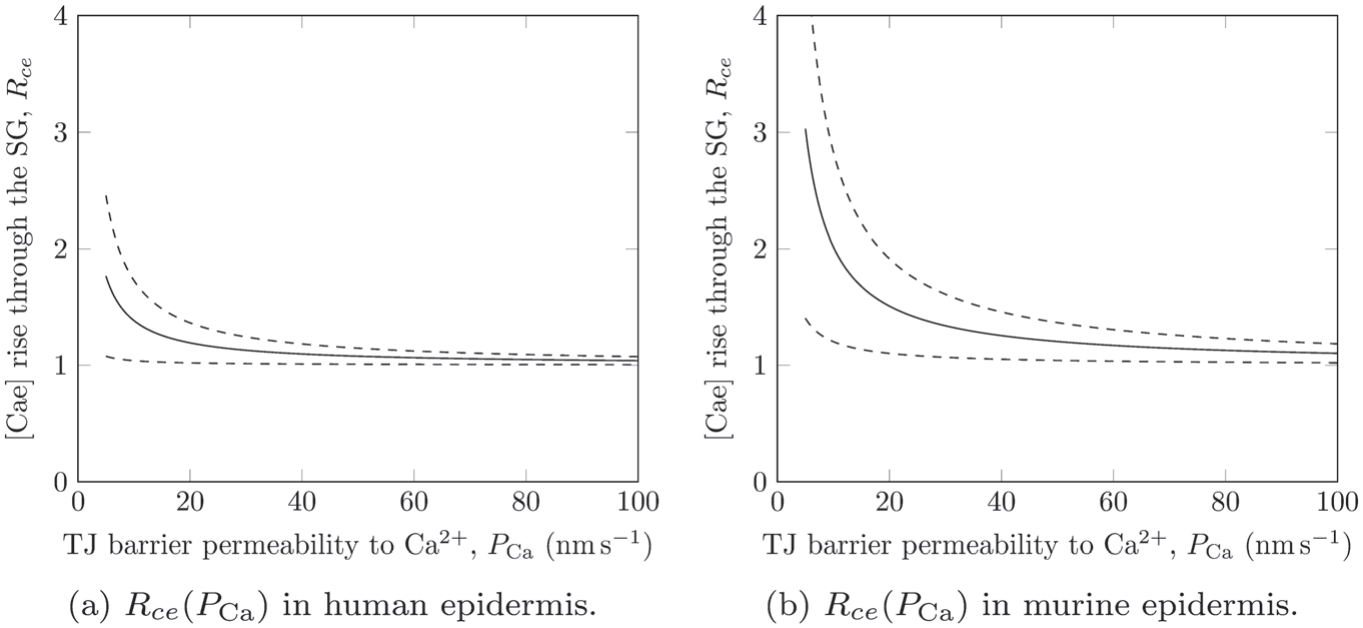
Extracellular calcium rise through the SG vs TJ permeability to calcium predicted by our model. The solid and dashed lines represent the mean values and uncertainty bounds (± SD) respectively.

Figs. 5a and 5b show the relationships between the rise in extracellular calcium through the SG and the permeability of the TJ barrier there, for human and murine epidermis respectively, that were predicted by our model using equations (22) and (23). Results are only shown for *P*_Ca_ ≥ 5 nm s^−1^ in order to satisfy applicability condition (17). As indicated by equation (22), each of these plots is characterised by one parameter *P*_0_ which depends on the epidermal keratinocyte velocity and calcium profiles; to construct Figs. 5a and 5b we obtained *P*_0_ = 3.8±3.2 nm s^−1^ and *P*_0_ = 10±8 nm s^−1^ for human and murine epidermis respectively. From these values, we calculated the permeability of the TJ barrier by assuming that the extracellular calcium concentration rises by at least 50% across the SG (i.e. *R*_*ce*_ = 1.5), based on experimental data for extracellular calcium distributions (see S1 Table). This calculation yielded TJ barrier permeabilities to calcium ions of *P*_Ca_ < 15 nm s^−1^ for human epidermis and *P*_Ca_ < 37 nm s^−1^ for murine epidermis.

### Extracellular and intracellular calcium profiles

**Figure 6:**
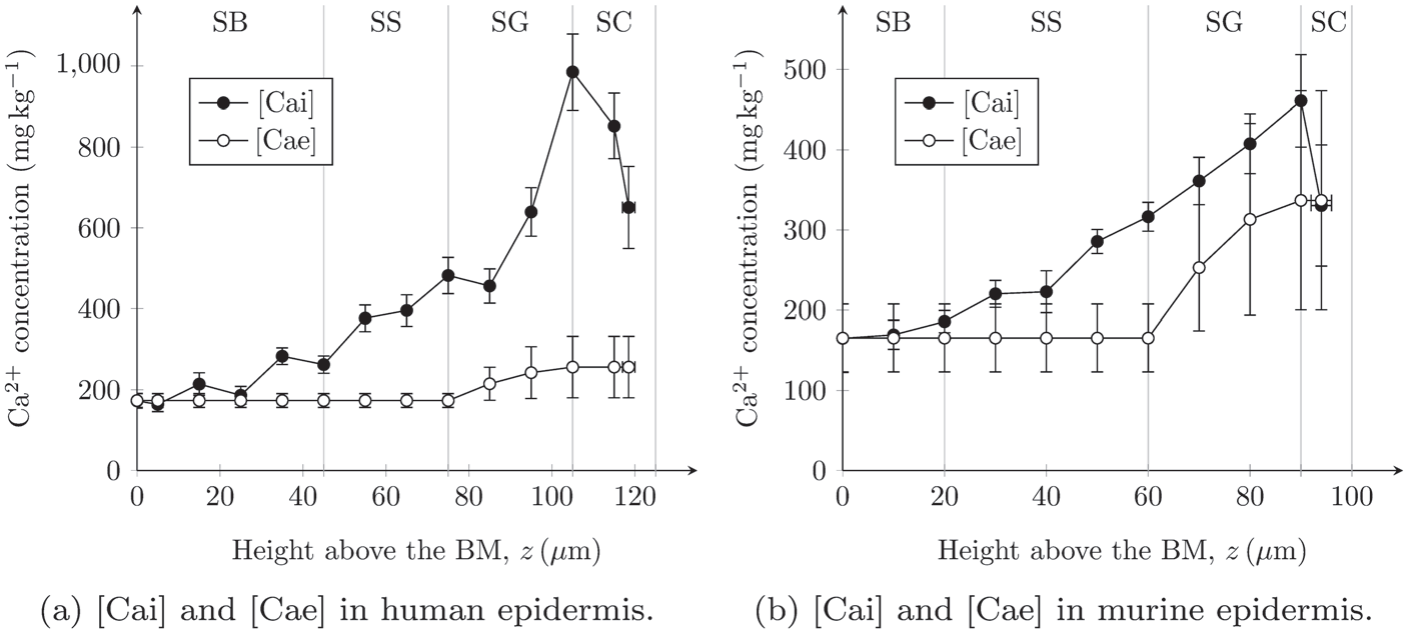
Physical intracellular ([Cai]) and extracellular ([Cae]) epidermal calcium profiles predicted by our model. These profiles are calculated from experimental total calcium profiles reported in [26, 28]. [Cae] profiles are shown for TJ barriers that yield a calcium rise through the SG of *R*_*ce*_ ≈ 1.5: (a) *P*_Ca_ = 8 nm s^−1^ for human epidermis and (b) *P*_Ca_ = 20 nm s^−1^ for murine epidermis.

Extracellular and intracellular epidermal calcium profiles, predicted from total calcium profiles *ρ*(*z*) and keratinocyte velocity profiles *u*_*i*_(*z*) using the equations of our model, are shown in Figs. 6a and 6b for human and murine epidermis respectively. The intracellular calcium profiles *ρ*_*ci*_(*z*) were nearly identical to the experimental total calcium profiles [26, 28] from which they were calculated. The extracellular calcium profiles *ρ*_*ce*_(*z*), calculated using equation (20), possessed constant concentration in the SB and SS due to rapid diffusion of this calcium throughout the ECF, and a rise through the SG due to the presence of TJs (see Fig. 2). In Figs. 6a and 6b we chose the permeability of the TJ barrier to calcium as *P*_Ca_ = 8 nm s^−1^ and *P*_Ca_ = 20 nm s^−1^ for human and murine epidermis respectively, as these values yielded a calcium rise through the SG of *R*_*ce*_ ≈ 1.5 in qualitative agreement with the experimental data (S1 Table). These values of TJ permeability barrier (8 nm s^−1^ for human epidermis and 20 nm s^−1^ for murine epidermis) also clearly satisfy the previously stated inequalities of *P*_Ca_ < 15 nm s^−1^ for human epidermis and *P*_Ca_ < 37 nm s^−1^ for murine epidermis.

**Figure 7:**
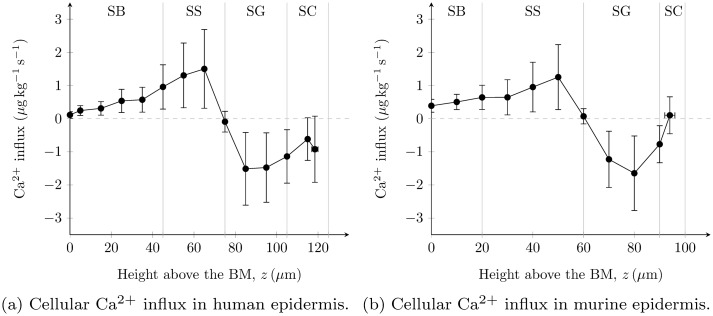
Keratinocyte calcium influx profiles *g*(*z*) in the epidermis predicted by our model. These profiles are calculated from experimental total calcium profiles reported in [26, 28].

Patterns of calcium exchange *g*(*z*) between keratinocytes and the ECF, predicted using equation (1b), are shown in Figs. 7a and 7b for human and murine epidermis respectively. In both plots, a distinct switch in calcium exchange from cellular influx (positive) to outflux (negative) was predicted at the SS-SG boundary, in agreement with our theory (Fig. 2).

## Discussion

In this paper we investigated the hypothesis that the intracellular and extracellular epidermal calcium profiles in unwounded skin are attributed to three key mechanisms: (1) the primary SC barrier which selectively allows water but not calcium to leave the epidermis [35], (2) progressive intracellular calcium accumulation through the lower epidermal sublayers [36] followed by a phenotypic switch at the SS-SG boundary to expulsion of intracellular calcium to the ECF above this boundary [23], and (3) reduced diffusion of extracellular calcium ions in the SG due to the secondary TJ barrier [43] which together with the aforementioned expulsion of calcium from intracellular stores causes the extracellular calcium concentration to become elevated towards the skin surface [44, 46]. This hypothesis was formulated in a mathematical model (described in Materials and Methods) that predicts intracellular and extracellular calcium profiles in human and murine epidermis (Fig. 6) which agree well with semi-quantitative experimental data available for these profiles [32–34].

We first parameterised the keratinocyte velocity profiles in human and murine epidermis, which is a requirement for the proper investigation of intracellular calcium dynamics. The calculation of these velocity profiles improves over our previous model [23] by including consideration of the slower cycling stem cell subpopulation of the SB [60] and the keratinocyte volume changes through the SS [11], and validating the velocity profiles against several sources of experimental data for keratinocyte transit times in the SB (human only) and the three suprabasal sublayers (SS, SG and SC).

The presence of stem cells in interfollicular epidermis is currently a hotly debated topic [6, 8]. Stem cells have little effect on the keratinocyte velocity profiles and subsequent calculations due to their small potential occupancy of the SB (1–10%, [64]), but their inclusion in the present model is advantageous as it allows validation of these profiles against transit times in the SB. Although our model assumed that the traditional two progenitor theory holds, it can be reduced to the single progenitor theory by setting *θ* = 0, in which case *s*_1_ is the proliferation rate of these progenitors.

The validation of keratinocyte velocity profiles against epidermal transit time data (Fig. 3) was made somewhat difficult by the uncertainty in both our predicted velocity profiles and the data. Despite this, the validation clearly supported the modification of one of our parameters, the volume change in keratinocytes through the SG for human epidermis (*V*_2_), from its value used in our previous model of unwounded epidermis (*R* = 1−*V*_2_, [23]). Our results suggested that keratinocytes in human epidermis may reduce their volume by approximately 10-fold during terminal differentiation and that this reduction may be even larger in murine epidermis.

We next investigated the effect of the permeability of the TJ barrier to calcium ions, *P*_Ca_, on the extracellular calcium distribution. Our model predictions of *P*_Ca_ carry large uncertainty due to the cumulative uncertainty in all parameters used to calculate them, and are only applicable if *P*_Ca_ is significantly greater than O(1 nm s^−1)^. Despite these limitations, we found that a value of *P*_Ca_ that is less than 15 nm s^−1^ for human epidermis and less than 37 nm s^−1^ for murine epidermis is sufficient to cause the extracellular calcium distribution to rise by at least 50% across the SG, which is a typical pattern seen in the experimental semi-quantitative calcium profiles measured using ion capture cytochemistry [32–34]. Kirschner *et al.* [45] recently reported that the permeability of the TJ barrier to calcium ions in cultured primary human keratinocytes was 40–80 nm s^−1^ within 1–4 days after a switch to high calcium medium (this switch is the key step in triggering keratinocytes to stratify *in vitro*[2]). These larger experimentally-found values of *P*_Ca_, which indicate a reduced TJ barrier to calcium ions, may be attributable to the impaired barrier formation demonstrated by cultured keratinocytes compared to native skin [33].

To further elucidate this point, the transepithelial resistance (TER) of the TJ barrier in the submerged human keratinocytes reported by Kirschner *et al.*[45] reached a steady-state value of ∼ 150 Ω cm^2^ after 4 days. In contrast, Sun *et al.* [71] and Petrova *et al.* [72] reported that the TER of the TJ barrier in human epidermal equivalents grown at an air-liquid interface (which yields a better representation of native epidermis than submerged keratinocytes [73]) rose to over 1000 Ω cm^2^ prior to formation of the lipid barrier. TER is the most common experimental measure of TJ barrier permeability [74], and is inversely related to it [75]. These considerations together suggest that the permeability of the TJ barrier should be less in native epidermis than in submerged keratinocytes grown *in vitro*. This agrees with our model prediction of a TJ barrier permeability to calcium ions in human epidermis that is less than the TJ barrier permeability to calcium ions experimentally observed in cultured human keratinocytes [45].

Finally, we calculated profiles of intracellular calcium, extracellular calcium and the exchange between these two (Figs. 6 and 7), from experimentally-reported total calcium profiles for human epidermis [28] and murine epidermis [26]. For the calculation of extracellular calcium profiles, we set the value of the calcium permeability of the epidermal TJ barrier so that it approximates an extracellular calcium concentration rise of 50% through the SG. The resulting profiles (Fig. 6) indicate that the physical intracellular calcium concentration is typically greater than the physical extracellular calcium concentration. Bearing in mind that intracellular and extracellular calcium are present in cells and ECF which occupy ≥ 93% and ≤ 7% of the epidermal volume respectively [36, 40], our model clearly predicts that intracellular calcium is the main source of the epidermal calcium profile [23].

The predicted pattern of calcium exchange between keratinocytes and the ECF (Figs. 6a and 6b) is significantly modified from our previous calculations of this pattern (Figs. 4c and 4d in [23]), due to the improved parameterisations used here for the keratinocyte volume changes through the SS and the SG, the former of which was assumed to be negligible in our previous models [23, 46]. The updated predictions cast doubt over the assertions in [23] that calcium influx is constant in the SB and SS and that there is a calcium influx peak in the lower SG potentially due to loss of plasma membrane Ca^2+^-ATPase [76]. However, the improved parameterisations confirmed the key finding of [23] that a change in calcium exchange from cellular influx to outflux actively regulates the epidermal calcium profile. The present theoretical work provides stronger evidence that this active regulation is caused by a phenotypic switch located at the SS-SG boundary (Fig. 7). The origin of this distinct switch in calcium exchange is currently being investigated with time-dependent continuum models developed by members of our research group [77].

Whilst our quantitative theory is able to predict the key features of intracellular and extracellular calcium profiles in unwounded epidermis, it has some potential weaknesses. We have assumed that the SC and TJ barriers are inert entities which regulate the epidermal calcium profile without any existing feedback processes, which is reasonable for considering unwounded epidermis as it represents a steady state condition. However, the formation of these barriers is likely to be dependent both on each other [78] and on the presence of the local calcium concentration [79, 80]. Hence this model cannot be immediately extended to consider temporal dynamics of wounded skin without specifying additional assumptions about the effects of epidermal calcium on the TJ and SC barriers. This is especially important since the rapid secretion by keratinocytes of lamellar bodies (the precursor to lipids that form the “mortar” component of the SC barrier) following barrier disruption is primarily controlled by calcium ions in the SG [13]. Whilst our conceptual model provides a feasible explanation for the formation of the calcium profile, especially as model parameters were obtained from experimental data, we cannot rule out the possibility of the contribution to this profile from other factors, such as the lipid barrier [78], electrophoresis [81], or binding of calcium to molecules such as profilaggrin [82]. In addition, if the factors that contribute substantially to the epidermal calcium profile occur on length scales of cells or smaller, our mathematical treatment of the epidermis as a porous medium may not be appropriate, and individual cell-based models (e.g. [83, 84]) are more suitable.

Our estimates of the TJ barrier permeability to calcium may require revision if the width of this barrier is larger or smaller than the SG. The effective TJ barrier may be larger than the SG if the TJ-like structures observed in the SC [67] reduce the extracellular calcium diffusion rate sufficiently there to yield protrusion of the extracellular calcium rise into the lower SC. On the other hand, the width of the TJ barrier may be smaller than the thickness of the SG, as recent experiments in mouse ear epidermis have suggested that only the TJs forming apically between the second of three cell monolayers of the SG are primarily responsible for its barrier [85]. Future experimental work may resolve this question about the localisation of TJ barrier function.

The investigations of the TJ barrier with our model were also limited to values for its permeability to calcium that satisfy inequality (17), which mathematically states the assumption that the TJ barrier permeability is significantly larger than the local ECF velocity in the absence of TJs. ECF flow is likely to be important for maintaining healthy unwounded epidermis, as occlusion of wounded skin by a vapour-permeable dressing (which permits low rates of transcutaneous water movement) is an adequate substitute for the SC whilst a vapour-impermeable dressing is not [35]. Future direct measurements of the TJ barrier permeability to calcium ions in native epidermis will hopefully confirm the applicability of inequality (17) and our subsequent mathematical theory relating the TJ barrier permeability to the extracellular calcium profile.

In conclusion, we have proposed and mathematically investigated a theory of calcium profile formation in unwounded mammalian epidermis governed by: the impermeable barrier of the SC, TJ-limited calcium diffusion in the SG, and a phenotypic switch in calcium exchange between keratinocytes and ECF at the SS-SG boundary. Future experimental results gained from improved measurement techniques [39, 86] may refine the presented theory and reduce the uncertainty in our model predictions. There are many possibilities for future theoretical work, including the investigation of temporally changing epidermal states for which calcium plays a major role (e.g. wound healing [35], psoriasis [34], and stratification of keratinocyte cultures [2]), and the consideration of our proposed calcium kinetics in individual cell-based models of epidermal homeostasis [83]. We intend that this paper provides a conceptual and quantitative model for future experimental and theoretical research to examine, modify and update, as our understanding of epidermal calcium profile formation becomes increasingly advanced.

## Supporting Information

S1 Text Justification of parameter values. (PDF)

S1 Table Semi-quantitative extracellular epidermal calcium distributions, determined using ion capture cytochemistry. (PDF)
