# Correction: Towards a Quantitative Theory of Epidermal Calcium Profile Formation in Unwounded Skin

**DOI:** 10.1371/journal.pone.0123823

**Published:** 2015-03-30

**Authors:** The PLOS ONE Staff

There is an error in the first sentence of the fifth paragraph in the “Model domain and boundary conditions” section of the Materials and Methods. The correct sentence is: The calcium present in the epidermis originates from movement of fluids and calcium across the BM [59], which at steady state must therefore act as a source of extracellular calcium with constant and positive concentration ρ0.

In the “Model domain and boundary conditions” section of the Materials and Methods, there is an error in Equation 2E. Please view the complete, correct equation here:

$$\rho_{ce}\left(0\right)\;=\;\rho_{0}$$ (2e)
