# Supplementary material for: Towards a Quantitative Theory of Epidermal Calcium Profile Formation in Unwounded Skin
Source: PLoS One. 2015 Jan 27;10(1):e0116751. doi: 10.1371/journal.pone.0116751 (PMC4308082; doi:10.1371/journal.pone.0116751)
Supplement: S1 Text — (PDF) [file pone.0116751.s001.pdf]

Text S1: Justification of Parameter Values

In the manuscript, Table 1 lists the parameters used in this paper and their key references. Here, we present the justification of the parameter values, starting from the experimental literature and applying subsequent calculations where necessary.

S.1 Stem cell volume fraction of the SB, θ

Li *et al.* state that epidermal stem cells constitute 1-10% of the SB based on several *in vivo* studies [1]. A range for the stem cell volume fraction of the SB that fully encompasses these estimates is $\theta = 0.055 \pm 0.045$. We assume that this value of θ applies to both human and murine epidermis.

S.2 Heights of the epidermal sublayer boundaries above the BM, z_1, z_2, z_3, z_4, z_5

For the human epidermal calcium profile investigated, the total epidermal thickness z_5 is $125\,\mu$m [2], and the thicknesses of the SB, SS, SG and SC are approximately 30-40 μm, 30 μm, 30 μm and 20 μm respectively, although the thickness of the SB is difficult to estimate due to undulation of the BM [3]. Hence we assume that the estimates of SS, SG and SC thickness are more accurate than the estimate of SB thickness, and consecutive subtraction of these values from $z_5 = 125\,\mu$m yields $z_3 = 105\,\mu$m, $z_2 = 75\,\mu$m and $z_1 = 45\,\mu$m.

For the estimation of z_4, we subtract from z_5 literature-reported estimates of the SC thickness removed at which transepidermal water loss (TEWL) becomes large. Bashir *et al.* reports that TEWL is significant after 5-7 μm of human SC is removed [4], whilst Kalia *et al.* reports that removal of $\sim$8 μm causes two- to ten-fold increase in TEWL [5]. We combine these values to estimate the thickness of upper SC in human epidermis as $6.5{\pm}1.5\mu$m, and hence $z_4 = 118.5 \pm 1.5\,\mu$m.

For the murine epidermal calcium profile, all sublayer boundary heights are provided except z_4 [6]: $z_1 = 20\,\mu$m, $z_2 = 60\,\mu$m, $z_3 = 90\,\mu$m and $z_5 = 100\,\mu$m. In murine epidermis TEWL increases dramatically once 4-8 μm has been removed [7]. We therefore assume that the thickness of murine upper SC is $6 \pm 2\,\mu$m, and hence $z_4 = 94 \pm 2\,\mu$m.

S.3 Ratio of keratinocyte volumes SG:SB, V_1

For human epidermis, Bergstresser *et al.* reported the volumes of keratinocytes in basal and superficial layers, in six human subjects and three anatomical locations for each subject [8]. We assume that each of the 18 associated ratios of superficial to basal keratinocyte volume are a good approximation of the volume change of a keratinocyte during its passage through the SS. From the mean and standard deviation of these 18 ratios, we obtain $V_1 = 1.9 \pm 0.5$.

For murine epidermis, the volumes of keratinocytes in the basal and granular sublayers have been reported both by Rowden [9], and by Rodrigues and Maia Campos [10]. Ratios of granular to basal keratinocyte volume, calculated from these publications, are 4.2 and 1.4 respectively. We combine these values to obtain $V_1 = 2.8 \pm 1.4$.

S.4 Ratio of keratinocyte volumes SC:SG, V_2

For human epidermis, the original estimate of V_2 is based on the report of Norlén and Al-Amoudi that there is a reduction in cell volume between SG and SC keratinocytes from 700-900 μm^3 to 400-450 μm^3 [11], which corresponds to $V_2 = 0.54 \pm 0.10$. For murine epidermis, Allen and Potten report that mouse dorsum keratinocyte volume changes from 163 μm^3 at the SB to 31.1 μm^3 at the skin surface [12].

This corresponds to $V_1 \times V_2 = 0.1908$. Dividing this by our obtained value of murine $V_1 = 2.8 \pm 1.4$ yields $V_2 = 0.068 \pm 0.034$.

In our results we found that the estimate of human V_2 was questionable, due to its prediction of transit times through the SC that disagreed strongly with the experimental literature and its order of magnitude difference from the estimate of murine V_2 which predicted SC transit times that agreed more reasonably with the experimental literature. Hence, for the modified estimate of V_2 for human epidermis, we used the human $V_1 = 1.9 \pm 0.5$ obtained from [8] together with the murine $V_1 \times V_2 = 0.1908$ from [12] to obtain $V_1 = 0.100 \pm 0.026$.

S.5 Proliferation rate of stem cells in the SB, s_0

The stem cell cycle time is difficult to measure, but is suggested to be greater than 500 hours in human epidermis and approximately 200 hours in murine epidermis [13]. The stem cell proliferation rate can be obtained simply by inverting the cycle time. For simplicity we assume that the human and murine stem cycle times are equal to 500 hours and 200 hours respectively, and inversion immediately yields human $s_0 = 5.6 \times 10^{-7}\,\mathrm{s}^{-1}$ and murine $s_0 = 1.4 \times 10^{-6}\,\mathrm{s}^{-1}$.

S.6 Proliferation rate of TA cells in the SB, s_1

For both human and murine epidermis, the TA cell proliferation rate s_1 is calculated from literature-reported values of the mean proliferation rate in the SB, denoted here as s_μ, together with our found values for θ and s_0, via the equation

$$s_\mu = \theta s_0 + (1 - \theta) s_1. \tag{1}$$

For human epidermis, Castelijns *et al.* reported a mean cycle time of approximately ∼62.5 hours [14], which together with the growth fraction in the SB of 60% [15], yields an estimate of $s_\mu = 2.7 \times 10^{-6}\,\mathrm{s}^{-1}$. On the other hand, Iizuka reported that there are 27,000 cells and a birth rate of 1,246 cells per day in a $1\,\mathrm{mm}^2$ section of the proliferative compartment of human epidermis [15]. Dividing the birth rate by the number of cells yields an alternative estimate of $s_\mu = 5.3 \times 10^{-7}\,\mathrm{s}^{-1}$. Combining these two estimates, we choose $s_\mu = (1.6 \pm 1.1) \times 10^{-6}\,\mathrm{s}^{-1}$. Then, using equation (1) we obtain $s_1 = (1.7 \pm 1.1) \times 10^{-6}\,\mathrm{s}^{-1}$.

For murine epidermis, Potten reported that the cell production rate in murine epidermis varies from 0.55 to 1.42 cells per 100 basal cells per hour, depending on the anatomical location [16]. This corresponds to a mean proliferation rate in the SB of $s_\mu = (2.7 \pm 1.2) \times 10^{-6}\,\mathrm{s}^{-1}$. Then, using equation (1) we obtain $s_1 = (2.8 \pm 1.3) \times 10^{-6}\,\mathrm{s}^{-1}$.

S.7 Physical diffusion coefficient of calcium in the ECF, D_{Ca}

For both human and murine epidermis, we assume that the ECF is essentially water [17], and hence D_{Ca} is equal to the diffusion coefficient of calcium ions in water at skin temperature. We assume that the value of this diffusion coefficient is unaltered for the one-dimensional case, as our model considers only one spatial direction z perpendicular to the skin surface. The calculation of this coefficient from data in [18–20] together with the Stokes-Einstein equation [18] is detailed in Appendix A of our previous paper [21], and yields $D_{\mathrm{Ca}} = 1 \times 10^{-9}\,\mathrm{m}^{-2}\,\mathrm{s}^{-1}$.

S.8 Cell volume fraction in viable epidermis, ϕ_v

For human epidermis, Celli *et al.* reported that the cell volume fraction increases from 0.93 in the SB to 0.98 in the SG [22]. We combine these values to choose $\phi_v = 0.955 \pm 0.025$.

For murine epidermis, Elias and Leventhal reported that the ECF volume fraction, $1 - \phi_v$, is 0.5-1.0% in the SG [23]. We assume this value applies throughout the viable epidermis, and hence choose $\phi_v = 0.9925 \pm 0.0025$.

S.9 Ratio of the extracellular calcium distribution to its BM value, r

To calculate r for human and murine epidermis, we use data from the semi-quantitative extracellular calcium distributions shown in Table S1.

For human epidermis, we assume that the number of positive signs is proportional to the extracellular calcium level. From the use of this assumption on the data in [24] and [25], the mean extracellular calcium levels in human SB, SS, SG and lower SC are 2, 2, 2.75 and 2 (i.e. overall mean of 2.2), and the minimum and maximum reported extracellular calcium levels in the whole epidermis excluding the upper SC are 1 and 3 respectively. This data can be enclosed by an extracellular calcium level throughout the whole epidermis excluding the upper SC of 2.2±1.2, which written as a ratio of the mean extracellular calcium level in human SB, yields r =1.1±0.6.

For murine epidermis, we fit a five-point quantitative scale to the worded descriptors in [26]: 1 (very low), 2 (low), 3 (medium), 4 (high) and 5 (very high). Using this scale, the mean extracellular calcium levels in the SB, SS, SG and SC are 2, 1, 4 and 3 (i.e. overall mean of 2.5), and the minimum and maximum reported extracellular calcium levels in the whole epidermis excluding the upper SC are 1 and 4 respectively. This data can be enclosed by an extracellular calcium level throughout the whole epidermis excluding the upper SC of 2.5±1.5, which written as a ratio of the mean extracellular calcium level in murine SB, yields r =1.25±0.75.

References

1. Li A, Simmons P, Kaur P (1998) Identification and isolation of candidate human keratinocyte stem cells based on cell surface phenotype. P Natl Acad Sci USA 85: 3902-3907.

2. Behne M, Tu CL, Aronchik I, Epstein E, Bench G, et al. (2003) Human keratinocyte ATP2C1 localizes to the Golgi Ca^{2+} stores. J Invest Dermatol 121: 688-694.

3. Personal communications with T. M. Mauro and A. Celli (2013).

4. Bashir SJ, Chew AL, Anigbogu A, Dreher F, Maibach HI (2001) Physical and physiological effects of stratum corneum tape stripping. Skin Res Technol 7: 40-48.

5. Kalia YN, Alberti I, Sekkat N, Curdy C, Naik A, et al. (2000) Normalization of stratum corneum barrier function and transepidermal water loss *in vivo*. Pharm Res 17: 1148-1150.

6. Mauro T, Bench G, Sidderas-Haddad E, Feingold K, Elias P, et al. (1998) Acute barrier perturbation abolishes the Ca^{2+} and K^{+} gradients in murine epidermis: quantitative measurement using PIXE. J Invest Dermatol 111: 1198-1201.

7. Yow HN, Wu X, Routh AF, Guy RH (2009) Dye diffusion from microcapsules with different shell thickness into mammalian skin. Eur J Pharm Biopharm 72: 62-68.

8. Bergstresser PR, Pariser RJ, Taylor JR (1978) Counting and sizing of epidermal cells in normal human skin. J Invest Dermatol 70: 280-284.

9. Rowden G (1975) Ultrastructural studies of keratinized epithelia of the mouse. III. Determination of the volumes of nuclei and cytoplasm of cells in murine epidermis. J Invest Dermatol 64: 1-3.

10. Rodrigues LHT, Maia Campos PMBG (2002) Comparative study of the effects of cosmetic formulations with or without hydroxy acids on hairless mouse epidermis by histopathologic, morphometric, and stereologic evaluation. J Cosmet Sci 53: 269-282.

11. Norlén L, Al-Amoudi A (2004) Stratum corneum keratin structure, function, and formation: the cubic rod-packing and membrane templating model. J Invest Dermatol 123: 715-732.

12. Allen T, Potten C (1976) Ultrastructural site variations in mouse epidermal organization. J Cell Sci 21: 341-359.

13. Potten C, Booth C (2002) Keratinocyte stem cells: a commentary. J Invest Dermatol 119: 888-899.

14. Castelijns F, Ezendam J, Latijnhouwers M, Vlijmen-Willems IV, Zeeuwen P, et al. (1998) Epidermal cell kinetics by combining *in situ* hybridization and immunohistochemistry. Histochem J 30: 869-877.

15. Iizuka H (1994) Epidermal turnover time. J Dermatol Sci 8: 215-217.

16. Potten C (1975) Epidermal cell production rates. J Invest Dermatol 65: 488-500.

17. Halprin K, Ohkawara A (1967) Glucose entry into the human epidermis: II. The penetration of glucose into the human epidermis *in vitro*. J Invest Dermatol 49: 561-568.

18. Li YH, Gregory S (1974) Diffusion of ions in sea water and in deep-sea sediments. Geochim Cosmochim Ac 38: 703-714.

19. Kampmeyer P (1952) The temperature dependence of viscosity for water and mercury. J Appl Phys 23: 99-102.

20. Williams E, Heusch A, McCarthy P (2008) Thermal screening of facial skin arterial hot spots using non-contact infrared radiometry. Physiol Meas 29: 341-348.

21. Adams MP, Mallet DG, Pettet GJ (2012) Active regulation of the epidermal calcium profile. J Theor Biol 301: 112-121.

22. Celli A, Sanchez S, Behne M, Hazlett T, Gratton E, et al. (2010) The epidermal Ca^{2+} gradient: measurement using the phasor representation of fluorescent lifetime imaging. Biophys J 98: 911-921.

23. Elias P, Leventhal M (1979) Intercellular volume changes and cell surface expansion during cornification. Clin Res 27: 525A.

24. Menon G, Elias P (1991) Ultrastructural localization of calcium in psoriatic and normal human epidermis. Arch Dermatol 127: 57-63.

25. Vičanová J, Boelsma E, Mommaas A, Kempenaar J, Forslind B, et al. (1998) Normalization of epidermal calcium distribution profile in reconstructed human epidermis is related to improvement of terminal differentiation and stratum corneum barrier formation. J Invest Dermatol 111: 97-106.

26. Menon G, Grayson S, Elias P (1985) Ionic calcium reservoirs in mammalian epidermis: Ultrastructural localization by ion-capture cytochemistry. J Invest Dermatol 84: 508-512.
