# Supplementary material for: Towards a Quantitative Theory of Epidermal Calcium Profile Formation in Unwounded Skin
Source: PLoS One. 2015 Jan 27;10(1):e0116751. doi: 10.1371/journal.pone.0116751 (PMC4308082; doi:10.1371/journal.pone.0116751)
Supplement: S1 Table — (PDF) [file pone.0116751.s002.pdf]

Table S1. Semi-quantitative extracellular epidermal calcium distributions, determined using ion capture cytochemistry

**Extracellular Calcium Concentration**						**Species, Reference**
**SB**	**SS**	**Lower SG**	**Upper SG**	**Lower SC**	**Upper SC**	
++	++	+++	+++	+++	0	Human, [1]
++	++	++	+++	+	0	Human, [2]
Low	Very low	High	High	High-Low	Low	Mouse, [3]

References

1. Menon G, Elias P (1991) Ultrastructural localization of calcium in psoriatic and normal human epidermis. Arch Dermatol 127: 57-63.

2. Vičanová J, Boelsma E, Mommaas A, Kempenaar J, Forslind B, et al. (1998) Normalization of epidermal calcium distribution profile in reconstructed human epidermis is related to improvement of terminal differentiation and stratum corneum barrier formation. J Invest Dermatol 111: 97-106.

3. Menon G, Grayson S, Elias P (1985) Ionic calcium reservoirs in mammalian epidermis: Ultrastructural localization by ion-capture cytochemistry. J Invest Dermatol 84: 508-512.
